# Comparison of Mitochondrial Adenosine Triphosphate–Sensitive Potassium Channel High- vs Low-Affinity Sulfonylureas and Cardiovascular Outcomes in Patients With Type 2 Diabetes Treated With Metformin

**DOI:** 10.1001/jamanetworkopen.2022.45854

**Published:** 2022-12-09

**Authors:** Meng-Ting Wang, Hsueh-Yi Pan, Ya-Ling Huang, Li-Wei Wu, Pin-Chun Wang, Yu-Juei Hsu, Tzu-Chieh Lin, ChenWei Lin, Jyun-Heng Lai, Chien-Hsing Lee

**Affiliations:** 1 Department of Pharmacy, National Yang Ming Chiao Tung University, Taipei, Taiwan; 2 School of Pharmacy, National Defense Medical Center, Taipei, Taiwan; 3 Division of Nephrology, Department of Internal Medicine, Tri-Service General Hospital, National Defense Medical Center, Taipei, Taiwan; 4 College of Pharmacy, The University of Texas at Austin, Austin; 5 Division of Endocrinology and Metabolism, Department of Internal Medicine, Tri-Service General Hospital, National Defense Medical Center, Taipei, Taiwan

## Abstract

**Importance:**

Sulfonylureas are frequently used as add-on to metformin in type 2 diabetes (T2D), and individual sulfonylurea agents carry different risks of cardiovascular disease. Sulfonylureas’ different affinities to cardiac mitochondrial adenosine triphosphate–sensitive potassium (mitoK_ATP_) channels have been speculated to account for the intraclass difference in cardiovascular risk from in vitro and ex vivo studies; however, this hypothesis has not been assessed in a general population with diabetes receiving sulfonylureas added to metformin.

**Objective:**

To compare the risk of myocardial infarction (MI), ischemic stroke, or cardiovascular death in patients with T2D treated with mitoK_ATP_ channel high-affinity sulfonylureas and low-affinity sulfonylureas as add-on to metformin.

**Design, Setting, and Participants:**

This is a new-user, active-comparator, and propensity score–matched cohort study with analysis of the Taiwanese Diabetes Mellitus Health Database from 2006, to 2017. Data analysis was performed from August 2020 to July 2021.

**Exposures:**

Cardiac mitoK_ATP_ channel high-affinity (glyburide and glipizide) and low-affinity (gliclazide and glimepiride) sulfonylureas combined with metformin.

**Main Outcomes and Measures:**

Primary outcome was major adverse cardiovascular events (MACEs), a composite of cardiovascular death or hospitalization for either MI or ischemic stroke. Secondary outcomes included individual MACE components, heart failure, arrhythmia, all-cause mortality, and severe hypoglycemia. Cox proportional hazards models were used to estimate adjusted hazard ratios (aHRs).

**Results:**

Each sulfonylurea group comprised 53 714 patients (mean [SD] age, 54.7 [12.1] years; 31 962 men [59.5%]). MitoK_ATP_ channel high-affinity sulfonylureas vs low-affinity sulfonylureas when combined with metformin were associated with an increased risk of MACE (aHR, 1.18; 95% CI, 1.03-1.34), MI (aHR, 1.34; 95% CI, 1.04-1.73), all-cause mortality (aHR, 1.27; 95% CI, 1.03-1.57), and severe hypoglycemia (aHR, 1.82; 95% CI, 1.58-2.10), but not with increased risks of ischemic stroke, cardiovascular death, arrhythmia, and heart failure. The duration analyses revealed the highest MACE risk during 1 to 90 days after initiation of mitoK_ATP_ channel high-affinity sulfonylureas (aHR, 6.06; 95% CI, 4.86-7.55).

**Conclusions and Relevance:**

Use of mitoK_ATP_ channel high-affinity sulfonylureas vs low-affinity sulfonylureas was associated with an increased MACE risk in patients with T2D concomitantly receiving metformin, suggesting that high-affinity blockage of the mitoK_ATP_ channels could account for sulfonylurea-associated MACEs.

## Key Points

**Question:**

Is there an increased risk of major adverse cardiovascular events (MACEs) associated with use of cardiac mitochondrial adenosine triphosphate–sensitive potassium (mitoK_ATP_) channel high-affinity sulfonylureas (glyburide and glipizide) vs low-affinity sulfonylureas (gliclazide and glimepiride) combined with metformin in type 2 diabetes (T2D)?

**Findings:**

In this population-based, propensity score–matched cohort study, use of cardiac mitoK_ATP_ channel high-affinity sulfonylureas vs low-affinity sulfonylureas added to metformin was associated with a 1.18-fold increased MACE risk in patients with T2D.

**Meaning:**

These findings suggest that high-affinity blockage of sulfonylureas to cardiac mitoK_ATP_ channels may act as an important potential mechanism underlying sulfonylureas-associated MACEs when combined with metformin in T2D.

## Teaser

This cohort study evaluates the association between mitochondrial adenosine triphosphate–sensitive potassium (K_ATP_) channel high- vs low-affinity sulfonylureas and cardiovascular outcomes in patients with type 2 diabetes treated with metformin.

## Introduction

Diabetes is a prevalent disease that imposes an enormous burden on health worldwide.^1^ Diabetes currently affects more than 537 million adults, leads to 6.9 million deaths, and cost $US 966 billion in health care expenditures in 2021 worldwide.^1^ Major cardiovascular events (MACEs) are the major cause of mortality and morbidity in diabetes; for example, approximately one-third of all deaths in patients with diabetes were cardiovascular causes from 2010 to 2015 in the US.^2^ This highlights the importance of preventing MACEs in patients with diabetes.

Despite the availability of newer types of antidiabetic medications,^3^ sulfonylureas remain one of the most frequently prescribed classes of noninsulin antihyperglycemic agents, primarily owing to their low cost, established glucose-lowering efficacy, and long experience of clinical use.^4^ Sulfonylureas are the most prescribed antihyperglycemic medications after metformin.^5,6^ However, use of sulfonylureas is associated with an elevated risk of MACEs.^4^ Different individual sulfonylureas have been found to be associated with varying risks of cardiovascular diseases,^7,8^ suggesting a possible intraclass difference in cardiovascular risk. Although it has been investigated whether the specificity of sulfonylureas for pancreatic β-cells could account for these drugs’ associated cardiovascular risks,^9,10,11,12^ no studies have confirmed the proposed mechanism.

Blockage of cardiac mitochondrial adenosine triphosphate–sensitive potassium (mitoK_ATP_) by certain sulfonylureas may be an alternative mechanism underlying the intraclass difference in MACEs. Ischemic preconditioning is the most pivotal mechanism for cardiac protection and is involved in the opening of mitoK_ATP_ channels, which can be blocked by certain sulfonylureas, such as glyburide and glipizide.^13,14^ In vitro and ex vivo data have indicated that there were differential affinities to the mitoK_ATP_ channels among individual sulfonylureas,^15,16,17,18,19^ although this has not been translated to account for sulfonylurea-related adverse cardiovascular events in clinical settings, except for 1 observational study focusing on sulfonylurea monotherapy.^20^ As sulfonylureas are most used as add-on antidiabetic agents to metformin in management of type 2 diabetes (T2D), we aimed to assess whether use of cardiac mitoK_ATP_ channel high-affinity sulfonylureas (glyburide and glipizide) vs low-affinity sulfonylureas (gliclazide and glimepiride) when combined with metformin is associated with an increased risk of MACEs in a nationwide T2D population.

## Methods

### Study Design and Data Sources

This was a population-based, propensity score (PS)–matched cohort study using the Taiwan Diabetes Mellitus Health Database (DMHD) from January 1, 2006 to December 31, 2017. The DMHD comprises patient diagnoses, medical procedures, and prescription refill records of all patients with new diagnoses of diabetes under the Taiwanese universal national health insurance (NHI) program, with a coverage rate of greater than 99% of Taiwanese inhabitants. In the database, patients with new diabetes diagnoses were defined as those with at least 3 outpatient visits or 1 inpatient visit for diabetes in a given year, and they did not have previous visits with any diagnoses of diabetes preceding the first visit related to diabetes. The nationwide death registry and the Tri-Service General Hospital (TSGH) electronic medical records were linked to the DMHD to obtain the causes of death and glycated hemoglobin A_1c_ (HbA_1c_) levels, respectively. This study was approved by the institutional review board of TSGH, National Defense Medical Center and completed before the lead author became affiliated with the National Yang Ming Chiao Tung University. Written informed consent was waived because the study analyzed a deidentified database. The report of this study followed the Strengthening the Reporting of Observational Studies in Epidemiology (STROBE) reporting guideline.

### Study Population and Exposures

We identified a base cohort of patients with newly diagnosed diabetes who initiated metformin monotherapy, defined as those without any prescription refill records of any antidiabetic medications in the year before the monotherapy commencement, from January 1, 2007, to December 31, 2016. The base cohort was required to have no type 1 diagnosis codes and to continuously receive metformin monotherapy, allowing a 30-day grace period between refills. From the base cohort, we identified the study cohort of patients with an add-on of a cardiac mitoK_ATP_ channel high-affinity sulfonylurea (glyburide and glipizide) or an add-on low-affinity sulfonylurea (gliclazide and glimepiride) to their metformin monotherapy, and marked the first prescription date of add-on sulfonylureas as cohort entry date. Patients who initiated sulfonylurea and metformin on the same day were also included. The study cohort was required to be aged 20 or more years at cohort entry. The 4 examined individual sulfonylureas comprised more than 99% of the sulfonylureas combined with metformin in the study period. Patients were excluded if they received both types of sulfonylureas at cohort entry; had lack of 1-year continuous NHI enrollment; or had a hospitalization with a primary diagnosis of myocardial infarction (MI), ischemic stroke, or pregnancy in the year preceding cohort entry. eTable 1 in Supplement 1 details the disease and procedure codes used in defining the inclusion and exclusion criteria.

The 2 sulfonylurea groups were followed from the cohort entry date until the earliest of the following events: occurrence of a primary cardiovascular outcome (defined later in this study), NHI enrollment discontinuation, metformin-sulfonylurea treatment discontinuation, switch or add-on of other antidiabetic medications (except for switching within the same mitoK_ATP_ channel high-affinity or low-affinity sulfonylurea group), pregnancy, or December 31, 2017. Discontinuous use of sulfonylureas with metformin was determined according to the prescription refill records with more than a 30-day grace period. We extended the follow-up to 1 month after the dual therapy was discontinued to include the MACEs events that may have caused discontinuity in the dual therapy.

The PS, the probability of initiating mitoK_ATP_ channel high-affinity sulfonylureas, was estimated by fitting a multivariable logistic regression model, conditional on all factors listed in Table 1, to maintain comparability between groups. The 2 sulfonylurea groups were matched by the cohort entry date (within 90 days), adapted Diabetes Complications Severity Index (aDCSI; 0, 1, 2, and ≥3), number of metformin prescriptions between initial use of metformin and cohort entry date, and PS using the nearest neighboring matching scheme with a caliper width of 0.02 on the propensity-score scale. We additionally examined the effects of the average daily dose (<0.5, 0.5-1, >1 defended daily dose) and duration therapy (1-90, 91-180, 181-365, >365 days) of mitoK_ATP_ channel high-affinity sulfonylureas on the comparative outcomes.

**Table 1.** Characteristics of Users of Add-on MitoK_ATP_ Channel High-Affinity and Low-Affinity Sulfonylureas Before and After Matching in Patients With Diabetes

Characteristics^a^	Before matching			After matching		
	Patients, No. (%)		SMD^b^	Patients, No. (%)		SMD^b^
	MitoK_ATP_ channel high-affinity sulfonylureas (n = 54 411)	MitoK_ATP_ channel low-affinity sulfonylureas (n = 193 333)		MitoK_ATP_ channel high-affinity sulfonylureas (n = 53 714)	MitoK_ATP_ channel low-affinity sulfonylureas (n = 53 714)	
Age, mean (SD), y	54.8 (12.1)	54.4 (11.9)	0.035	54.7 (12.1)	54.6 (12.0)	0.006
Sex						
Female	22 132 (40.7)	80 265 (41.5)	0.017	21 833 (40.6)	21 671 (40.3)	0.006
Male	32 279 (59.3)	113 068 (58.5)	0.017	31 881 (59.4)	32 043 (59.7)	0.006
Metformin prescriptions between initial use of metformin and cohort entry date, mean (SD), No.	1.11 (2.90)	1.68 (3.55)	0.176	1.02 (2.70)	1.02 (2.70)	<0.001
Entry year						
2007	5954 (10.9)	12 823 (6.6)	0.150	5874 (10.9)	5910 (11.0)	0.002
2008	6611 (12.2)	15 181 (7.9)	0.142	6484 (12.1)	6417 (12.0)	0.004
2009	7683 (14.1)	18 061 (9.3)	0.147	7552 (14.1)	7614 (14.2)	0.003
2010	6630 (12.2)	19 356 (10.0)	0.069	6537 (12.2)	6565 (12.2)	0.002
2011	6310 (11.6)	19 190 (9.9)	0.054	6230 (11.6)	6206 (11.6)	0.001
2012	5631 (10.4)	20 939 (10.8)	0.016	5574 (10.4)	5616 (10.5)	0.003
2013	4987 (9.2)	21 675 (11.2)	0.068	4942 (9.2)	4917 (9.2)	0.002
2014	4226 (7.8)	22 198 (11.5)	0.127	4186 (7.8)	4156 (7.7)	0.002
2015	3499 (6.4)	22 974 (11.9)	0.192	3479 (6.5)	3486 (6.5)	0.001
2016	2880 (5.3)	20 936 (10.8)	0.206	2856 (5.3)	2827 (5.3)	0.002
Diabetes severity indicators						
Adapted diabetes complications severity index						
0	44 748 (82.2)	158 060 (81.8)	0.013	44 572 (83.0)	44 572 (83.0)	<0.001
1	6393 (11.8)	23 812 (12.3)	0.017	6214 (11.6)	6214 (11.6)	<0.001
2	2684 (4.9)	9484 (4.9)	0.001	2497 (4.7)	2497 (4.7)	<0.001
≥3	586 (1.1)	1977 (1.0)	0.005	431 (0.8)	431 (0.8)	<0.001
Metformin dose						
<1000 mg	20 634 (37.9)	59 693 (30.9)	0.145	20 237 (37.7)	19 822 (36.9)	0.016
1000-1499 mg	25 270 (46.4)	91 750 (47.5)	0.020	25 070 (46.7)	25 478 (47.4)	0.015
≥1500 mg	8507 (15.6)	41 890 (21.7)	0.158	8407 (15.7)	8414 (15.7)	<0.001
Measures of health care utilization						
Physician visits, No.						
Diabetes related						
First tertile	29 777 (54.7)	88 395 (45.7)	0.172	29 678 (55.3)	29 831 (55.5)	0.006
Second tertile	10 171 (18.7)	41 180 (21.3)	0.066	10 145 (18.9)	10 091 (18.8)	0.003
Third tertile	14 463 (26.6)	63 758 (33.0)	0.143	13 891 (25.9)	13 792 (25.7)	0.004
Non–diabetes related						
First tertile	17 814 (32.7)	62 506 (32.3)	0.009	17 719 (33.0)	18 343 (34.2)	0.025
Second tertile	18 171 (33.4)	66 702 (34.5)	0.023	17 981 (33.5)	17 755 (33.1)	0.009
Third tertile	18 426 (33.9)	64 125 (33.2)	0.015	18 014 (33.5)	17 616 (32.8)	0.016
Hospital admissions, No.						
Diabetes related						
0	53 118 (97.6)	188 743 (97.6)	<0.001	52 596 (97.9)	52 613 (98.0)	0.002
1	1091 (2.0)	3959 (2.1)	0.003	986 (1.8)	979 (1.8)	0.001
2	202 (0.4)	631 (0.3)	0.008	132 (0.3)	122 (0.2)	0.004
Non–diabetes related						
0	51 174 (94.1)	182 978 (94.6)	0.026	50 614 (94.2)	50 682 (94.4)	0.005
1	2491 (4.6)	7971 (4.1)	0.022	2396 (4.5)	2367 (4.4)	0.003
2	746 (1.4)	2384 (1.2)	0.012	704 (1.3)	665 (1.2)	0.006
Emergency department visits, No.						
Diabetes related						
0	53 454 (98.2)	189 675 (98.1)	0.010	52 830 (98.4)	52 882 (98.5)	0.008
1	752 (1.4)	3206 (1.7)	0.023	713 (1.3)	683 (1.3)	0.005
2	205 (0.4)	452 (0.2)	0.026	171 (0.3)	149 (0.3)	0.008
Non–diabetes related						
0	45 641 (83.9)	163 089 (84.4)	0.013	45 192 (84.1)	45 443 (84.6)	0.013
1	5892 (10.8)	21 316 (11.0)	0.006	5774 (10.8)	5580 (10.4)	0.012
2	2878 (5.3)	8928 (4.6)	0.031	2748 (5.1)	2691 (5.0)	0.005
Monthly income-based insurance premium, NTD						
First tertile	22 061 (40.6)	66 682 (34.5)	0.122	21 693 (40.4)	21 742 (40.5)	0.002
Second tertile	15 471 (28.4)	61 652 (31.9)	0.076	15 285 (28.5)	15 380 (28.6)	0.004
Third tertile	16 879 (31.0)	64 999 (33.6)	0.056	16 736 (31.2)	16 592 (30.9)	0.006
Hospital level						
No medical record	1769 (3.3)	4908 (2.5)	0.042	1769 (3.3)	1985 (3.7)	0.022
Academic medical centers	3599 (6.6)	14 758 (7.6)	0.040	3546 (6.6)	3445 (6.4)	0.008
Metropolitan hospitals	6649 (12.2)	23 782 (12.3)	0.002	6501 (12.1)	6368 (11.9)	0.008
Local community hospitals	5165 (9.5)	14 834 (7.7)	0.065	4915 (9.2)	4778 (8.9)	0.009
Physician clinics	37 229 (68.4)	135 051 (69.9)	0.031	36 983 (68.9)	37 138 (69.1)	0.006
Comorbidities						
Cardiovascular diseases						
Heart failure	1110 (2.0)	3510 (1.8)	0.016	1008 (1.9)	1003 (1.9)	0.001
Hypertension	21 205 (39.0)	79 077 (40.9)	0.040	20 726 (38.6)	20 070 (37.4)	0.025
Cerebrovascular disease	1328 (2.4)	4445 (2.3)	0.009	1239 (2.3)	1225 (2.3)	0.002
Ischemic heart disease	3885 (7.1)	14 049 (7.3)	0.007	3676 (6.8)	3768 (7.0)	0.007
Arrhythmia	111 (0.2)	325 (0.2)	0.008	93 (0.2)	82 (0.2)	0.005
Dyslipidemia	13 211 (24.3)	59 408 (30.7)	0.148	12 963 (24.1)	12 652 (23.6)	0.014
Peripheral arterial disease	621 (1.1)	2132 (1.1)	0.004	585 (1.1)	583 (1.1)	<0.001
Coronary revascularization	81 (0.2)	318 (0.2)	0.004	74 (0.1)	76 (0.1)	0.001
Cardiomyopathy	79 (0.2)	276 (0.1)	0.001	70 (0.1)	65 (0.1)	0.003
Venous thromboembolism	74 (0.1)	238 (0.1)	0.004	66 (0.1)	60 (0.1)	0.003
Pulmonary disease						
Asthma	1979 (3.6)	7180 (3.7)	0.004	1927 (3.6)	1882 (3.5)	0.005
Chronic obstructive pulmonary disease	1891 (3.5)	5582 (2.9)	0.033	1773 (3.3)	1731 (3.2)	0.040
Pneumonia	1219 (2.2)	4019 (2.1)	0.011	1145 (2.1)	1093 (2.0)	0.007
Mental disease						
Depression	1249 (2.3)	4511 (2.3)	0.003	1219 (2.3)	1183 (2.2)	0.005
Anxiety	3928 (7.2)	14 331 (7.4)	0.007	3854 (7.2)	3769 (7.0)	0.006
Schizophrenia	593 (1.1)	1805 (0.9)	0.016	565 (1.1)	515 (1.0)	0.010
Neurologic disorders						
Dementia	341 (0.6)	1048 (0.5)	0.011	313 (0.6)	298 (0.6)	0.004
Epilepsy	179 (0.3)	536 (0.3)	0.009	166 (0.3)	151 (0.3)	0.005
Bone and joint disorders						
Fracture	1774 (3.3)	6044 (3.1)	0.008	1721 (3.2)	1702 (3.2)	0.002
Osteoporosis	749 (1.4)	2455 (1.3)	0.009	724 (1.4)	728 (1.4)	0.001
Osteoarthritis	5645 (10.4)	19 712 (10.2)	0.006	5490 (10.2)	5353 (10.0)	0.008
Anemia	855 (1.6)	3380 (1.8)	0.014	823 (1.5)	830 (1.6)	0.001
Thyroid disease	1279 (2.4)	5484 (2.8)	0.031	1255 (2.3)	1208 (2.3)	0.006
Chronic liver disease	5418 (10.0)	19 718 (10.2)	0.008	5251 (9.8)	5115 (9.5)	0.009
Chronic kidney disease	1899 (3.5)	7917 (4.1)	0.032	1749 (3.3)	1752 (3.3)	<0.001
Obesity or weight gain	1471 (2.7)	4893 (2.5)	0.011	1441 (2.7)	1374 (2.6)	0.008
Tobacco-related	459 (0.8)	1910 (1.0)	0.015	452 (0.8)	461 (0.9)	0.002
Alcohol-related disorder	241 (0.4)	719 (0.4)	0.011	233 (0.4)	242 (0.5)	0.003
Hypokalemia	229 (0.4)	713 (0.4)	0.008	209 (0.4)	179 (0.3)	0.009
Hypoglycemia	149 (0.3)	663 (0.3)	0.012	142 (0.3)	143 (0.3)	<0.001
Autoimmune diseases	998 (1.8)	3255 (1.7)	0.011	955 (1.8)	969 (1.8)	0.002
Cancer	1733 (3.2)	5996 (3.1)	0.005	1670 (3.1)	1643 (3.1)	0.003
Comedication						
Cardiovascular medications						
Angiotensin-converting enzyme inhibitors	3987 (7.3)	13 286 (6.9)	0.018	3846 (7.2)	3781 (7.0)	0.005
Angiotensin receptor blockers	7515 (13.8)	35 144 (18.2)	0.121	7332 (13.7)	7005 (13.0)	0.018
α-Blockers	938 (1.7)	3048 (1.6)	0.012	898 (1.7)	855 (1.6)	0.006
β-Blockers	10 462 (19.2)	36 425 (18.8)	0.010	10 143 (18.9)	9865 (18.4)	0.013
Calcium channel blockers						
Dihydropyridines	13 706 (25.2)	49 949 (25.8)	0.015	13 370 (24.9)	12 968 (24.1)	0.017
Nondihydropyridines	1144 (2.1)	3838 (2.0)	0.008	1092 (2.0)	1066 (2.0)	0.003
Diuretics						
Thiazides	10 816 (19.9)	43 148 (22.3)	0.060	10 541 (19.6)	10 086 (18.8)	0.021
Loop	1754 (3.2)	5583 (2.9)	0.019	1643 (3.1)	1666 (3.1)	0.002
Potassium-sparing agents	1316 (2.4)	3681 (1.9)	0.035	1209 (2.3)	1206 (2.3)	<0.001
Antiplatelets	7037 (12.9)	24 716 (12.8)	0.004	6760 (12.6)	6579 (12.3)	0.010
Anticoagulants	391 (0.7)	1390 (0.7)	<0.001	358 (0.7)	373 (0.7)	0.003
Lipid-lowering agents						
Statins	5575 (10.3)	29 749 (15.4)	0.156	5417 (10.1)	5116 (9.5)	0.019
Others	857 (1.6)	3684 (1.9)	0.025	842 (1.6)	860 (1.6)	0.003
Nitrates	1163 (2.1)	3659 (1.9)	0.017	1070 (2.0)	1028 (1.9)	0.006
Antiarrhythmic agents	460 (0.9)	1456 (0.8)	0.010	420 (0.8)	406 (0.8)	0.003
Digoxin	531 (1.0)	1626 (0.8)	0.014	485 (0.9)	496 (0.9)	0.002
Anti-inflammatory agents						
Nonsteroidal anti-inflammatory drugs	31 071 (57.1)	111 250 (57.5)	0.009	30 642 (57.1)	30 321 (56.5)	0.012
Steroids	8980 (16.5)	31 548 (16.3)	0.005	8779 (16.3)	8542 (15.9)	0.012
Potassium channel opener (nicorandil)	301 (0.6)	1395 (0.7)	0.021	287 (0.5)	274 (0.5)	0.003
Inhibitors of mitochondrial permeability transition pore	8872 (16.3)	31 692 (16.4)	0.002	8679 (16.2)	8495 (15.8)	0.009
Proton pump inhibitors	1537 (2.8)	5744 (3.0)	0.009	1485 (2.8)	1421 (2.7)	0.007
Anticonvulsants	1779 (3.3)	6704 (3.5)	0.011	1712 (3.2)	1643 (3.1)	0.007
Antidepressants	2941 (5.4)	10 562 (5.5)	0.003	2864 (5.3)	2813 (5.2)	0.004
Antipsychotics	3502 (6.4)	11 724 (6.1)	0.015	3388 (6.3)	3123 (5.8)	0.021

Abbreviations: mitoK_ATP_, mitochondrial adenosine triphosphate–sensitive potassium; NTD, New Taiwan dollar; SMD, standardized mean difference.

^a^ All comorbidities, diabetes severity indicators, and adjusted Diabetes Complications Severity Index were measured in the year preceding the cohort entry date; comedications were measured 6 months before the cohort entry date.

^b^ SMD greater than 0.1 represents meaningful differences between 2 groups.

### Outcome Definition

The primary outcome was 3-point composite outcome of MACEs, including hospitalization with any diagnosis of MI, ischemic stroke, or cardiovascular death. The positive predictive values of the coding algorithms (eTable 1 in Supplement 1) for identifying the MI and ischemic stroke events in the database were at least 88%.^21,22^ Secondary outcomes were the individual components of the 3-point MACEs, heart failure, arrhythmia, all-cause mortality, and hypoglycemia, as detailed in eTable 1 in Supplement 1.

### Baseline Covariates

We assessed patient demographic and clinical characteristics in the year preceding the cohort entry, including age, sex, proxies of diabetes severity (eg, aDCSI and metformin daily dose), comorbidities (eg, chronic kidney disease), and health care utilizations. Comedications (eg, cardiovascular medications) were evaluated in the previous 6 months before cohort entry.

### Statistical Analysis

#### Main Analysis

Baseline characteristics were compared using the standardized difference, where a magnitude greater than 0.1 indicated an imbalance between groups. The Kaplan-Meier method with a log-rank test was used to compare the cumulative incidence of primary and secondary outcomes. Cox proportional hazards models with 95% CIs were used to estimate the hazard ratios (HRs) for each outcome, and the proportionality assumption was assessed through Schoenfeld residuals, with all assumptions being met. All analyses were also adjusted for PS deciles after the matching process to mitigate any residual confounding. Statistical significance was defined as a 2-sided value of *P* < .05. Data cleaning and statistical analyses were performed using SAS statistical software version 9.4 (SAS Institute). Data were analyzed from August 2020 to July 2021.

#### Sensitivity and Subgroup Analysis

Multiple predetermined sensitivity analyses were conducted. First, we performed the PS calibration analysis,^23^ which combined PS and regression calibration, to address the unavailability of HbA_1c_ in the DMHD by using HbA_1c_ data obtained from a tertiary medical center, detailed in eMethods in Supplement 1. Second, unmeasured confounding was addressed through the rule-out approach^24^ and high-dimensional PS-matched analyses (detailed in eMethods in Supplement 1).^25^ Third, the grace period used for determining continuous treatment was shortened to 14 days to address any exposure misclassification. Fourth, we performed an intention-to-treat analysis without considering treatment discontinuation or switching, where follow-up was set as 1 year to mitigate potential informative censoring. Fifth, we considered noncardiovascular death as a competing event for the primary outcome using the Fine and Gray method. Sixth, all patients were restricted to those with a medication possession ratio of 80% or greater to maintain adequate medication adherence.^26^ Seventh, we performed a negative outcome of diabetic retinopathy analysis as there is no evidence suggesting differential risks of the eye disease between the 2 types of sulfonylureas. Eighth, we adjusted for hypoglycemic events measured during follow-up to rule out the possibility that the observed MACE risk was mediated through hypoglycemia. Ninth, cardiovascular death in the MACE outcome was redefined as a primary cause of MI or ischemic stroke. Tenth, an inverse probability of treatment weighting using PS was performed to avoid reduction in sample sizes.^27^ We additionally performed subgroup analyses according to sulfonylurea’s pancreas specificity (pancreas-specificity sulfonylureas, glipizide vs gliclazide; pancreas-nonspecific sulfonylureas, glyburide vs glimepiride), with re-estimated PS.

## Results

**Figure 1.**
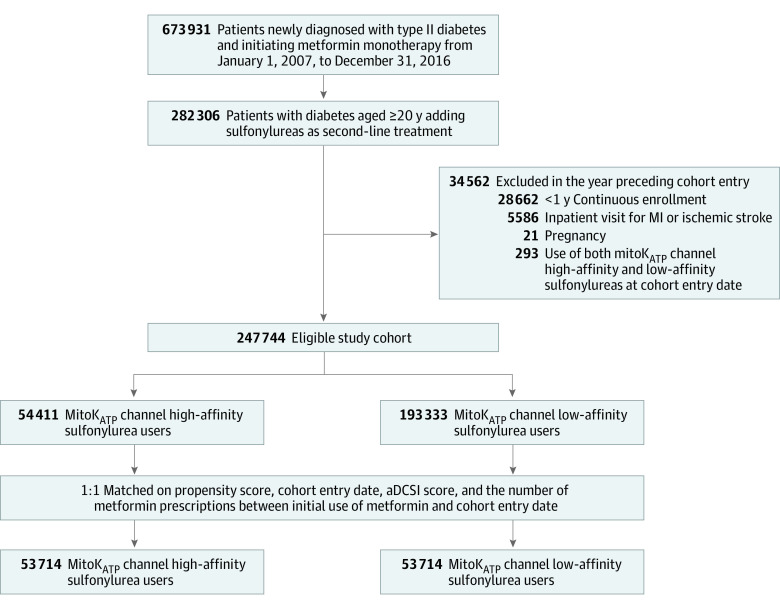
Flowchart Showing the Selection of the Base and Study Cohorts aDCSI, adapted Diabetes Complications Severity Index; MI, myocardial infarction; mitoK_ATP_, mitochondrial adenosine triphosphate–sensitive potassium.

A total of 247 744 patients with T2D met the inclusion and exclusion criteria, among whom after 1:1 matching 53 714 pairs of new users of mitoK_ATP_ channel low-affinity sulfonylureas and high-affinity sulfonylureas in combination with metformin were identified. Overall mean (SD) patient age was 54.7 (12.1) years, and 31 962 (59.5%) were men (Figure 1). The mean (SD) follow-up duration was 10.2 (16.3) months (corresponding to 44 790 person-years) and 14.0 (20.5) months (61 816 person-years) in the mitoK_ATP_ channel high-affinity and low-affinity sulfonylurea group, respectively. Most patients in both affinity groups were censored during the follow-up primarily owing to treatment discontinuation (29 054 [54.1%] and 31 671 [59.0%], respectively) or treatment switching (15 953 [29.7%] and 13 286 [24.7%], respectively), with the differences between groups across all types of censoring no greater than 5% (eTable 2 in Supplement 1). In our study, glyburide and glimepiride comprised 76.8% and 74.9% in the cardiac mitoK_ATP_ channel high-affinity sulfonylurea and in low-affinity sulfonylurea groups, respectively.

As shown in Table 1, although most baseline characteristics were similar between groups before matching, more patients in the mitoK_ATP_ channel low-affinity sulfonylurea group entered the study cohort in later years, had diagnoses of dyslipidemia, and received angiotensin receptor blockers and statins than did the high-affinity sulfonylurea group. All characteristics were well-balanced after matching.

A total of 444 and 484 MACEs occurred in the mitoK_ATP_ channel high-affinity and in the low-affinity sulfonylurea group, respectively, during follow-up, with corresponding incidence rates per 100 person-years of 0.99 (95% CI, 0.90-1.09) and 0.78 (95% CI, 0.72-0.86) (Table 2). The cumulative incidence rates of the examined outcomes are shown in eFigures 1 and 2 in Supplement 1.

**Table 2.** Risk of Major Adverse Cardiovascular Events Between Add-on MitoK_ATP_ Channel High-Affinity Sulfonylurea and MitoK_ATP_ Channel Low-Affinity Sulfonylurea

Outcomes	MitoK_ATP_ channel high-affinity sulfonylureas (n = 53 714)			MitoK_ATP_ channel low-affinity sulfonylureas (n = 53 714)			HR (95% CI)	aHR (95% CI)^a^
	Events, No.	Total No. of person-years	Incidence rate per 100 person-years (95% CI)	Events, No.	Total No. of person-years	Incidence rate per 100 person-years (95% CI)		
Primary outcome, 3-point MACE^b^	444	44 790	0.99 (0.90-1.09)	484	61 816	0.78 (0.72-0.86)	1.17 (1.03-1.34)	1.18 (1.03-1.34)
Secondary outcomes								
Myocardial infarction	118	44 909	0.26 (0.22-0.31)	119	62 009	0.19 (0.16-0.23)	1.34 (1.04-1.73)	1.34 (1.04-1.73)
Ischemic stroke	295	44 847	0.66 (0.59-0.74)	330	61 879	0.53 (0.48-0.59)	1.12 (0.96-1.31)	1.12 (0.96-1.31)
Cardiovascular death^c^	38	44 964	0.08 (0.06-0.12)	39	62 069	0.06 (0.05-0.09)	1.32 (0.85-2.07)	1.32 (0.85-2.07)
Arrhythmia	81	44 901	0.18 (0.15-0.22)	89	61 983	0.14 (0.12-0.18)	1.20 (0.89-1.63)	1.21 (0.89-1.63)
Heart failure	59	44 952	0.13 (0.10-0.17)	55	62 045	0.09 (0.07-0.12)	1.35 (0.93-1.95)	1.35 (0.93-1.95)
All-cause mortality	169	44 961	0.38 (0.32-0.44)	174	62 066	0.28 (0.24-0.33)	1.27 (1.02-1.57)	1.27 (1.03-1.57)
Severe hypoglycemia	485	44 750	1.08 (0.99-1.19)	330	61 889	0.53 (0.48-0.59)	1.82 (1.58-2.09)	1.82 (1.58-2.10)

Abbreviations: aHR, adjusted hazard ratio; HR, hazard ratio; MACE, major adverse cardiovascular events; mitoK_ATP_, mitochondrial adenosine triphosphate–sensitive potassium.

^a^ Adjusted for the deciles of propensity scores.

^b^ Three-point MACE includes myocardial infarction, ischemic stroke, and cardiovascular death.

^c^ Cardiovascular death was defined as death due to all cardiovascular diseases.

MitoK_ATP_ channel high-affinity sulfonylureas were associated with a 1.18-fold (95% CI, 1.03-1.34) increased risk of MACE compared with mitoK_ATP_ channel low-affinity sulfonylureas in combination with metformin (Table 2). Additionally, use of mitoK_ATP_ channel high-affinity sulfonylureas vs low-affinity sulfonylureas was associated with an increased risk of MI (adjusted HR [aHR], 1.34; 95% CI, 1.04-1.73), all-cause mortality (aHR, 1.27; 95% CI, 1.03-1.57), and severe hypoglycemia (aHR, 1.82; 95% CI, 1.58-2.10) but was not associated with higher risks of ischemic stroke, cardiovascular death, arrhythmia, and heart failure in patients with T2D concurrently receiving metformin.

The MACE risk varied by duration and dose of mitoK_ATP_ channel high-affinity sulfonylurea therapy (Table 3). The duration of mitoK_ATP_ channel high-affinity sulfonylurea use was inversely associated with the MACE risk, with the highest increased risk confined to 1 to 90 days of the sulfonylurea therapy (aHR, 6.06; 95% CI, 4.86-7.55). Among the 3 dose strata, only the use of mitoK_ATP_ channel high-affinity sulfonylureas greater than 1 defined daily dose was associated with an increased risk of MACEs (aHR, 1.43; 95% CI, 1.10-1.85). eTable 3 in Supplement 1 presents the absolute risks.

**Table 3.** Risk of Major Adverse Cardiovascular Events With Different Doses and Durations of Add-on MitoK_ATP_ Channel High-Affinity Sulfonylurea Therapy Compared With Any Use of MitoK_ATP_ Channel Low-Affinity Sulfonylurea^a^

Dose and duration	Events, No.	Total No. of person-years	Incidence rate per 100 person-years (95% CI)	HR (95% CI)	aHR (95% CI)^b^
MitoK_ATP_ channel low-affinity sulfonylureas	484	61 816	0.78 (0.72-0.86)	1 [Reference]	1 [Reference]
Cumulative duration of add-on mitoK_ATP_ channel high-affinity sulfonylurea					
MitoK_ATP_ channel high-affinity sulfonylureas, d					
1-90	202	2824	7.15 (6.23-8.21)	6.09 (4.89-7.59)	6.06 (4.86-7.55)
91-180	45	3652	1.23 (0.92-1.65)	1.39 (0.999-1.92)	1.38 (0.996-1.92)
181-365	49	5371	0.91 (0.69-1.21)	1.17 (0.86-1.59)	1.17 (0.86-1.59)
>365	148	32 942	0.45 (0.38-0.53)	0.59 (0.49-0.71)	0.59 (0.49-0.72)
Average daily dose of add-on mitoK_ATP_ channel high-affinity sulfonylurea					
MitoK_ATP_ channel high-affinity sulfonylureas, defined daily dose					
<0.5	182	18 372	0.99 (0.86-1.15)	1.13 (0.95-1.34)	1.12 (0.95-1.33)
0.5-1	197	20 872	0.94 (0.82-1.09)	1.15 (0.97-1.36)	1.16 (0.98-1.37)
>1	65	5546	1.17 (0.92-1.49)	1.42 (1.09-1.83)	1.43 (1.10-1.85)

Abbreviations: aHR, adjusted hazard ratio; HR, hazard ratio; MACE, major adverse cardiovascular events; mitoK_ATP_, mitochondrial adenosine triphosphate–sensitive potassium.

^a^ Three-point MACE includes myocardial infarction, ischemic stroke, and cardiovascular death.

^b^ Adjusted for the deciles of propensity scores.

**Figure 2.**
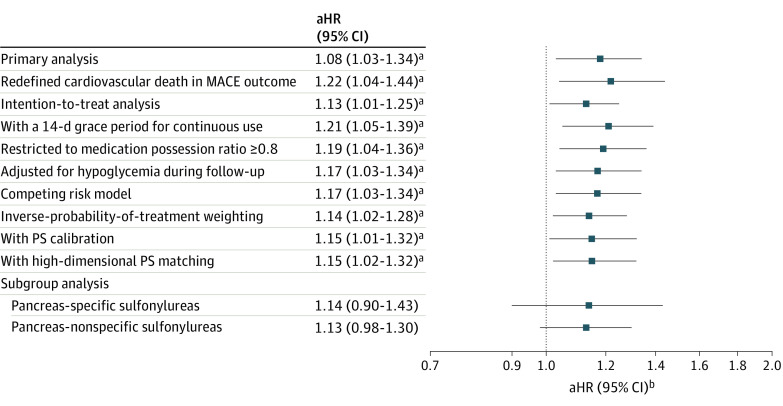
Forest Plots of Sensitivity and Subgroup Analyses aHR, adjusted hazard ratio; MACE, major adverse cardiovascular events; PS, propensity score. ^a^*P *value is less than .05. ^b^The deciles of PS were adjusted for.

The main findings remained consistent across all sensitivity analyses, such as when considering baseline HbA_1c_ in estimating the PS and using intention-to-treat analyses (Figure 2). Additionally, an unmeasured confounder was unlikely to fully explain the main findings with the rule-out approach (eFigure 3 in Supplement 1). Furthermore, the negative outcome analysis revealed a null association (aHR, 1.01; 95% CI, 0.90-1.14), as expected. The HRs were not materially changed after stratifying by the pancreas specificity of sulfonylureas.

## Discussion

The overall findings suggested that use of mitoK_ATP_ channel high-affinity sulfonylureas vs low-affinity sulfonylureas in combination with metformin was associated with an increased MACE risk in a nationwide T2D population. The increased MACE risk was primarily associated with hospitalization for MI and was particularly elevated with mitoK_ATP_ channel high-affinity sulfonylureas used within 90 days of initiation and at a high daily dose. The main findings were consistent across multiple sensitivity analyses. Our findings suggest that high affinity to cardiac mitoK_ATP_ channels is associated with sulfonylurea-associated cardiovascular risk when concomitantly used with metformin in patients with T2D.

In accordance with this current finding, a recently published observational study,^20^ reported a 21% (aHR, 1.21; 95% CI, 1.03-1.44) increased risk of 3-point MACEs associated with mitoK_ATP_ channel high-affinity sulfonylureas vs low-affinity sulfonylureas although the prior study focused on sulfonylurea monotherapy, and the generalizability of the reported data might be limited, given that sulfonylureas are most prescribed as an add-on medication to metformin in clinical settings. Conversely, the 2 studies observed discrepant secondary outcome findings, such as a substantial excess in cardiovascular death in the prior study as opposed to no statistically increased cardiovascular mortality for use of mitoK_ATP_ channel high-affinity sulfonylureas compared with low-affinity sulfonylureas in this current study. Although there may be differences in study cohort characteristics and diabetes severity between the 2 studies, use of metformin may at least partly account for the contradictory data. Despite inconsistent findings,^28^ metformin could be associated with reduced risk of cardiovascular-related events and cardiovascular mortality in patients with T2D,^29,30^ and this benefit may offset or modify the risk of the cardiovascular events associated with use of mitoK_ATP_ channel high-affinity sulfonylureas. On the other hand, the Action in Diabetes and Vascular Disease: Preterax and Diamicron MR-Controlled Evaluation (ADVANCE) and The Cardiovascular Outcome Study of Linagliptin vs Glimepiride in Type 2 Diabetes (CAROLINA) trials found that the mitoK_ATP_ channel low-affinity sulfonylurea gliclazide modified release vs other antidiabetic medication and linagliptin vs the mitoK_ATP_ channel low-affinity sulfonylureas glimepiride, respectively, had comparable outcomes on the risk of MACEs.^31,32^ Although not directly comparable to this current study, the data from the 2 large prospective studies^31,32^ indirectly support our reported data.

Overall, our findings are supported by biological plausibility. In vitro and animal data revealed that certain sulfonylureas could jeopardize or abolish ischemic preconditioning, the self-cardiac protection mechanism for minimizing a potentially lethal ischemic result, through antagonism of cardiac mitoK_ATP_ channels, which could subsequently cause larger infarct sizes or lead to death during an acute ischemic event.^16,33,34^ Our findings are in line with these preclinical data, offering insights into the cardiovascular safety of sulfonylureas combined with metformin in patients with T2D.

The findings on the hypoglycemia outcome are supported by the prior literature revealing that glyburide had the highest risk of hypoglycemia, followed by glimepiride and glipizide.^35,36^ In our study, glyburide and glimepiride comprised 76.8% and 74.9% in the cardiac mitoK_ATP_ channel high-affinity sulfonylurea and in low-affinity sulfonylurea groups, respectively. This could explain why there was a higher risk of hypoglycemia in the high-affinity sulfonylurea users compared with the low-affinity sulfonylurea users. Additionally, the main findings persisted after adjusting for occurrence of hypoglycemia during follow-up, which may rule out the possibility that hypoglycemia acts as an intermediator for our observed associations.

Our data have implications in prescribing practice and comparative safety research of antidiabetic agents in T2D. As cardiac mitoK_ATP_ channel low-affinity sulfonylureas were found to have lower risks of both 3-point MACEs and hypoglycemia, as compared with cardiac mitoK_ATP_ channel high-affinity sulfonylureas, we recommend low-affinity sulfonylureas over high-affinity sulfonylureas in cases where sulfonylurea treatment is considered for patients with T2D. This recommendation about the choice of sulfonylureas is of great clinical importance, given that sulfonylureas are one of the most used antidiabetic drugs after metformin in current clinical settings, despite the presence of newer antidiabetic medications. Additionally, some newer antidiabetic agents have been compared to sulfonylureas as a class regarding the risk of cardiovascular diseases,^37,38^ but the reported comparative data may have been associated with the compositions of varying mitoK_ATP_ channel affinity sulfonylurea. Future comparative studies involving sulfonylureas are suggested to take the affinity of sulfonylureas to cardiac mitoK_ATP_ channels into account.

### Strengths and Limitations

This study had several strengths. To our knowledge, this is the first study that showed pharmacological differences in sulfonylureas combined with metformin regarding their differential affinity to cardiac mitoK_ATP_ channels, which could account for the sulfonylurea intraclass difference in the risk of MACEs among sulfonylurea agents. The inclusion of a nationwide diabetic population with the most used sulfonylurea regimen as add-on therapy to metformin substantially increases the generalizability of our findings. The validity of the composite outcome of 3-point MACEs is expected to be high, as the coding algorithms for identifying hospitalization for MI or ischemic stroke were validated with high accuracy,^21,22^ and cardiovascular deaths were determined through a nationwide death registry.

Several limitations of this study merit discussion. First, our study is potentially subjected to confounding by indication or disease severity. We attempted to mitigate this potential confounding by maintaining a balanced average daily dose of metformin and all proxies of diabetes severity between the 2 groups. Second, unmeasured confounders, such as smoking, could possibly confound the reported findings; however, we used an active-comparator design, and reached consistent findings when using the high-dimensional–PS approach. Third, the adopted as-treated exposure analyses were prone to potential informative censoring; nevertheless, the alternative intention-to-treat analysis yielded similar results as the main findings. Fourth, the long-term effect of cardiac mitoK_ATP_ channel low-affinity sulfonylureas and high-affinity sulfonylureas on cardiovascular safety could not be assessed due to the fairly short follow-up period. The observed short-term use of sulfonylureas combined with metformin, however, reflects clinical medication use among patients with T2D. Fifth, the adoption of a PS-matching approach led to reductions in sample sizes, and we alternatively adopted the inverse probability of treatment weighting approach which maintained the original sample sizes and led to the consistent results. Sixth, we may not have sufficient statistical power in secondary and subgroup analyses including duration analyses, which needs to be taken into consideration when interpreting the findings.

## Conclusions

MitoK_ATP_ channel high-affinity sulfonylureas vs low-affinity sulfonylureas, when combined with metformin, were associated with an increased risk of MACE, suggesting that the high-affinity blockage of cardiac mitoK_ATP_ channels may act as an important determinant of sulfonylurea-related adverse cardiovascular events in patients with T2D.

## Notes

Supplement 1. **eMethods. **The Propensity Score Calibration Analysis
eReferences
**eTable 1.** Operational Definitions for the Adopted Exclusion Criteria, Outcomes, Comorbidities, and Comedications **eTable 2.** The Mean Duration and the Reasons for Truncation During Follow-Up for Add-On Mitochondrial KATP Channel High-Affinity and Low-Affinity Sulfonylurea Groups, by Outcomes **eTable 3.** Number Needed to Harm for Add-On Mitochondrial K_ATP_ Channel High-Affinity and Low-Affinity Sulfonylureas In Patients Continuously Receiving Metformin Monotherapy **eFigure 1.** Kaplan-Meier Survival Curves of 3-Point Major Adverse Cardiovascular Events (A), Myocardial Infarction (B), Ischemic Stroke (C), and Cardiovascular Death (D) Between Metformin Users With Add-On Mitochondrial K_ATP_ Channel High-Affinity Sulfonylurea and Mitochondrial K_ATP_ Channel Low-Affinity Sulfonylurea **eFigure 2.** Kaplan-Meier Survival Curves of Arrhythmia (A), Heart Failure (B), All-Cause Mortality (C), and Severe Hypoglycemia (D) Between Metformin Users With Add-On Mitochondrial K_ATP_ Channel High-Affinity Sulfonylurea and Mitochondrial K_ATP_ Channel Low-Affinity Sulfonylurea **eFigure 3.** Employment of the Rule-Out Approach to Assess the Impact of Unmeasured Confounding on the Main Findings

Supplement 2.
Data Sharing Statement
