# Supplementary material for: Comparison of Mitochondrial Adenosine Triphosphate–Sensitive Potassium Channel High- vs Low-Affinity Sulfonylureas and Cardiovascular Outcomes in Patients With Type 2 Diabetes Treated With Metformin
Source: JAMA Netw Open. 2022 Dec 9;5(12):e2245854. doi: 10.1001/jamanetworkopen.2022.45854 (PMC9856426; doi:10.1001/jamanetworkopen.2022.45854)
Supplement: Supplement 1. — eMethods. The Propensity Score Calibration Analysis eReferences eTable 1. Operational Definitions for the Adopted Exclusion Criteria, Outcomes, Comorbidities, and Comedications eTable 2. The Mean Duration and the Reasons for Truncation During Follow-Up for Add-On Mitochondrial KATP Channel High-Affinity and Low-Affinity Sulfonylurea Groups, by Outcomes eTable 3. Number Needed to Harm for Add-On Mitochondrial KATP Channel High-Affinity and Low-Affinity Sulfonylureas In Patients Continuously Receiving Metformin Monotherapy eFigure 1. Kaplan-Meier Survival Curves of 3-Point Major Adverse Cardiovascular Events (A), Myocardial Infarction (B), Ischemic Stroke (C), and Cardiovascular Death (D) Between Metformin Users With Add-On Mitochondrial KATP Channel High-Affinity Sulfonylurea and Mitochondrial KATP Channel Low-Affinity Sulfonylurea eFigure 2. Kaplan-Meier Survival Curves of Arrhythmia (A), Heart Failure (B), All-Cause Mortality (C), and Severe Hypoglycemia (D) Between Metformin Users With Add-On Mitochondrial KATP Channel High-Affinity Sulfonylurea and Mitochondrial KATP Channel Low-Affinity Sulfonylurea eFigure 3. Employment of the Rule-Out Approach to Assess the Impact of Unmeasured Confounding on the Main Findings [file jamanetwopen-e2245854-s001.pdf]

Supplemental Online Content

Wang MT, Pan HY, Huang YL, et al. Comparison of mitochondrial adenosine triphosphate–sensitive potassium channel high- vs low-affinity sulfonylureas and cardiovascular outcomes in patients with type 2 diabetes treated with metformin. *JAMA Netw Open*. 2022;5(12):e2245854. doi:10.1001/jamanetworkopen.2022.45854

eMethods. The Propensity Score Calibration Analysis
eReferences
eTable 1. Operational Definitions for the Adopted Exclusion Criteria, Outcomes, Comorbidities, and Comedications
eTable 2. The Mean Duration and the Reasons for Truncation During Follow-Up for Add-On Mitochondrial KATP Channel High-Affinity and Low-Affinity Sulfonylurea Groups, by Outcomes
eTable 3. Number Needed to Harm for Add-On Mitochondrial K_{ATP} Channel-High Affinity and Low-Affinity Sulfonylureas In Patients Continuously Receiving Metformin Monotherapy
eFigure 1. Kaplan-Meier Survival Curves of 3-Point Major Adverse Cardiovascular Events (A), Myocardial Infarction (B), Ischemic Stroke (C), and Cardiovascular Death (D) Between Metformin Users With Add-On Mitochondrial K_{ATP} Channel High-Affinity Sulfonylurea and Mitochondrial K_{ATP} Channel Low-Affinity Sulfonylurea
eFigure 2. Kaplan-Meier Survival Curves of Arrhythmia (A), Heart Failure (B), All-Cause Mortality (C), and Severe Hypoglycemia (D) Between Metformin Users With Add-On Mitochondrial K_{ATP} Channel High-Affinity Sulfonylurea and Mitochondrial K_{ATP} Channel Low-Affinity Sulfonylurea
eFigure 3. Employment of the Rule-Out Approach to Assess the Impact of Unmeasured Confounding on the Main Findings

This supplemental material has been provided by the authors to give readers additional information about their work.

© 2022 Wang MT et al. *JAMA Network Open.*

eMethods. The Propensity Score Calibration Analysis

To address the lack of data available on HbA_{1c} levels in the analyzed Taiwan Diabetes Mellitus Health Database (DMHD), a propensity score calibration (PSC) approach was performed to assess any measurement error in the PS of our main study and observe for possible effects of the unobserved confounder.[1,2] This PSC analysis required the study population of the main study, which consisted of 53,714 users of add-on mitoK_{ATP} channel-high affinity and -low affinity sulfonylureas, respectively, as well as the study population of a separate validation study. The additional information on HbA_{1c} levels was assessed from the electronic medical records of Tri-service General Hospital, a tertiary medical center in Taiwan, the data of which are managed by the Tri-service General Hospital Integrated Database Center (IRDC, TSGH). By linking the electronic healthcare records from the IRDC, TSGH with the DMHD that was used in the main study, we obtained a subgroup of study population (i.e., study calibration population) that included the HbA_{1c} level, consisting of 3,024 patients with diabetes.

We estimated two different PSs in the validation study using similar logistic regression models. The first PS used a multiple logistic regression model that had the same set of predictors as the main study and hence corresponds to the error-prone PS model. The second PS, referred to as the gold standard PS, was also estimated on the same predictors used in the error-prone model but included additional information on the HbA_{1c} level. We then fitted a linear regression model regressing the gold standard PS with the error-prone PS. The resulting linear equation was then applied in the study population to transform the original PS into the gold standard scores.

The employed high-dimensional propensity score analysis

To account for unmeasured confounding that could impact our findings, high-dimensional propensity score (HdPS) analysis was conducted by using a computerized algorithm to automatically select covariates from multidimensional datasets within the examined database.[3,4] Each patient with add-on mitoK_{ATP} channel-high affinity sulfonylurea was matched to one patient with add-on mitoK_{ATP} channel-low affinity sulfonylurea on the estimated HdPS. A total of 53,746 pairs of patients were matched after employment of the HdPS matching scheme. The algorithms for estimating the HdPS are detailed as follows:

© 2022 Wang MT et al. *JAMA Network Open.*

1) Independent variables were automatically selected from nine data dimensions. The various data dimensions describe different aspects of care and were defined as records of disease diagnoses (3-digit ICD-9-CM/ICD-10-CM codes), medical procedures (5-digit ICD-9/ICD-10 procedure codes), and prescribed medications (5-digit ATC codes) in outpatient, emergency care, and inpatient claims records, respectively.

2) We then identified variables that should not be part of the covariate adjustment, specifically variables that are low in prevalence, defined as appearing in fewer than 100 patients, and those that are instrumental. The variables in the nine data dimensions were prioritized based on its potential to bias the exposure-outcome relation under the main study. Hence, variables were excluded if it had a strong association with the exposure of interest but a weak association with the outcome of interest, defined as $|\log(OR_{CE})| > 1.5$ and $|\log(RR_{CD})| < 0.5$,[5] or if it had low prevalence.[4,6] The OR_{CE} and RR_{CD} represent the odds ratio of an exposure associated with a variable and the relative risk of an outcome associated with a variable, respectively.

3) All variables from the dimensions were sorted and ranked based on their prevalence, which was measured as the proportion of patients having at least one record of a specific code for the variable of interest. The top 200 most prevalent variables were identified in each data dimension.

4) A total of 1,800 variables were selected from the nine data dimension combined. In order to reduce the total number of variables, we prioritized them based on the absolute value of the logarithm of the multiplicative bias term ($Bias_M$), using the following formula:

   $Bias_M = [Pc_1(RR_{CD}-1)+1]/ [Pc_0(RR_{CD}-1)+1]$,[4]

   where Pc_1 and Pc_0 are the proportion of patients for whom the presence of a variable is observed in the treatment and control group, respectively. RR_{CD} stands for the relative risk of the outcome associated with a variable.

   After prioritization based on the magnitude of $Bias_M$ among the 1,800 variables, 500 empirical variables were selected for inclusion in the PS model.

5) In addition to the 500 empirical variables, multiple pre-specified variables were forced into

© 2022 Wang MT et al. *JAMA Network Open.*

the HdPS model, including age at cohort entry, sex, calendar year of entry, and adapted Diabetes Complications Severity Index (aDCSI; 0, 1, 2, and ≥ 3). After HdPS estimation, users of add-on mitoK$_{ATP}$ channel-high affinity sulfonylurea were matched with users of add-on mitoK$_{ATP}$ channel-low affinity sulfonylurea using a greedy, nearest neighbor 1:1 matching algorithm without replacement. Matching was based on the logit of the HdPS using a caliper width equal to 0.2 of the standard deviation.

6) After HdPS matching, 53,746 pairs were kept, and all variables were balanced among these patients. Poisson models and Cox proportional hazard regression models were then employed to estimate the incidence rates and hazard ratios of the outcomes with 95% confidence intervals.

eReferences

1. Stürmer T, Schneeweiss S, Avorn J, Glynn RJ. Adjusting effect estimates for unmeasured confounding with validation data using propensity score calibration. *Am J Epidemiol.* 2005;162(3):279-289.
2. Stürmer T, Schneeweiss S, Rothman KJ, Avorn J, Glynn RJ. Performance of propensity score calibration-a simulation study. *Am J Epidemiol.* 2007;165(10):1110-1118.
3. Guertin JR, Rahme E, Dormuth CR, LeLorier J. Head to head comparison of the propensity score and the high-dimensional propensity score matching methods. *BMC Med Res Methodol.* 2016;16:22.
4. Schneeweiss S, Rassen JA, Glynn RJ, Avorn J, Mogun H, Brookhart MA. High-dimensional propensity score adjustment in studies of treatment effects using health care claims data. *Epidemiology*. 2009;20(4):512.
5. Schneeweiss S, Eddings W, Glynn RJ, Patorno E, Rassen J, Franklin JM. Variable selection for confounding adjustment in high-dimensional covariate spaces when analyzing healthcare databases. *Epidemiology*. 2017;28(2):237-248.
6. Rassen JA, Glynn RJ, Brookhart MA, Schneeweiss S. Covariate selection in high-dimensional propensity score analyses of treatment effects in small samples. *Am J Epidemiol.* 2011;173(12):1404-1413.

eTable 1. Operational Definitions for the Adopted Exclusion Criteria, Outcomes, Comorbidities, and Comedications

**Inclusion criteria**	**Diagnosis codes**
Diabetes mellitus	ICD-9: 250; ICD-10: E10-E14
No Type I diabetes mellitus	ICD-9: 250.x1, 250.x3; ICD-10: E10
**Exclusion criteria**	**Diagnosis codes**
Pregnancy	ICD-9: 630-677; ICD-10: O00-O99, O9A
Myocardial infarction	ICD-9-CM code: 410; ICD-10-CM code: I21
Ischemic stroke	ICD-9-CM code: 433, 434; ICD-10-CM code: I63-I66
**Outcome definition**	**Diagnosis codes**
Major cardiovascular events	
Myocardial infarction	ICD-9-CM code: 410; ICD-10-CM code: I21
Ischemic stroke	ICD-9-CM code: 433, 434; ICD-10-CM code: I63-I66
Cardiovascular death	ICD-9-CM code: 390-459; ICD-10-CM code: I00-I99
Cardiac arrhythmia	ICD-9-CM code: 427; ICD-10-CM code: I47-49
Hypoglycemia	ICD-9-CM code: 251.0, 251.1, 251.2, and 250.8 (ICD-9-CM code 250.8 accompanied by the following diagnosis codes that represent other diseases were excluded: 259.8, 272.7, 681, 682, 686.9, 707, 709.3, 730.0, 730.1, 730.2, and 731.8); ICD-10-CM code: E08.641, E08.649, E09.641, E09.649, E11.63, E11.641, E11.649, E13.63, E13.641, E13.649, E14.63, E15, E16.0, E16.1, E16.2
**Comorbidities**	**Diagnosis codes**
CV diseases	
Heart failure	ICD-9-CM code: 402.x1, 404.x1, 404.x3, 428; ICD-10-CM code: I11.0, I13.0, I13.2, I50
Hypertension	ICD-9-CM code: 401-405; ICD-10-CM code: I10-15
Cerebrovascular disease	ICD-9-CM code: 430-432, 435-438; ICD-10-CM code: I60-I62, I67-I69, G45, G46
Ischemic heart disease	ICD-9-CM code: 411-414; ICD-10-CM code: I20, I22-I25
Arrhythmia	ICD-9-CM code: 427; ICD-10-CM code: I47-49
Dyslipidemia	ICD-9-CM code: 272; ICD-10-CM code: E71.30, E75.21, E75.22, E75.24, E75.3, E75.5, E75.6, E77, E78, E88.1, E88.2, E88.89
Peripheral vascular disease	ICD-9-CM code: 440, 443.9; ICD-10-CM code: I70, I75, I73.9
Coronary revascularization	ICD-9-CM procedure code: 00.66, 17.55, 36.0, 36.1, 36.2; ICD-10-PCS code: 0210093, 0210098, 0210099, 021009C, 021009F, 021009W, 02100A3, 02100A8, 02100A9, 02100AC, 02100AF, 02100AW, 02100J3, 02100J8, 02100J9, 02100JC, 02100JF, 02100JW, 02100K3, 02100K8, 02100K9, 02100KC, 02100KF, 02100KW, 02100Z3, 02100Z8, 02100Z9, 02100ZC, 02100ZF, 0210493, 0210498, 0210499, 021049C, 021049F, 021049W, 02104A3, 02104A8, 02104A9, 02104AC, 02104AF, 02104AW, 02104J3, 02104J8, 02104J9, 02104JC, 02104JF, 02104JW, 02104K3, 02104K8, 02104K9, 02104KC, 02104KF, 02104KW, 02104Z3, 02104Z8, 02104Z9, 02104ZC, 02104ZF, 0211093, 0211098, 0211099, 021109C, 021109F, 021109W, 02110A3, 02110A8, 02110A9, 02110AC, 02110AF, 02110AW, 02110J3, 02110J8, 02110J9, 02110JC, 02110JF, 02110JW, 02110K3, 02110K8, 02110K9, 02110KC, 02110KF, 02110KW, 02110Z3, 02110Z8, 02110Z9, 02110ZC, 02110ZF, 0211493, 0211498, 0211499, 021149C, 021149F, 021149W, 02114A3, 02114A8, 02114A9, 02114AC, 02114AF, 02114AW, 02114J3, 02114J8, 02114J9, 02114JC, 02114JF, 02114JW, 02114K3, 02114K8, 02114K9, 02114KC, 02114KF, 02114KW, 02114Z3, 02114Z8, 02114Z9, 02114ZC, 02114ZF, 0212093, 0212098, 0212099, 021209C, 021209F, 021209W, 02120A3, 02120A8, 02120A9, 02120AC, 02120AF, 02120AW, 02120J3, 02120J8, 02120J9, 02120JC, 02120JF, 02120JW, 02120K3, 02120K8, 02120K9, 02120KC, 02120KF, 02120KW, 02120Z3, 02120Z8, 02120Z9, 02120ZC, 02120ZF, 0212493, 0212498, 0212499, 021249C, 021249F, 021249W, 02124A3, 02124A8, 02124A9, 02124AC, 02124AF, 02124AW, 02124J3, 02124J8, 02124J9, 02124JC, 02124JF, 02124JW, 02124K3, 02124K8, 02124K9, 02124KC, 02124KF, 02124KW, 02124Z3, 02124Z8, 02124Z9, 02124ZC, 02124ZF, 0213093, 0213098, 0213099, 021309C, 021309F, 021309W, 02130A3, 02130A8, 02130A9, 02130AC, 02130AF, 02130AW, 02130J3, 02130J8, 02130J9, 02130JC, 02130JF, 02130JW, 02130K3, 02130K8, 02130K9, 02130KC, 02130KF, 02130KW, 02130Z3, 02130Z8, 02130Z9, 02130ZC, 02130ZF, 0213493, 0213498, 0213499, 021349C, 021349F, 021349W, 02134A3, 02134A8, 02134A9, 02134AC, 02134AF, 02134AW, 02134J3, 02134J8, 02134J9, 02134JC, 02134JF, 02134JW, 02134K3, 02134K8, 02134K9, 02134KC, 02134KF, 02134KW, 02134Z3, 02134Z8, 02134Z9, 02134ZC,

© 2022 Wang MT et al. *JAMA Network Open.*

	02134ZF, 021K0Z8, 021K0Z9, 021K0ZC, 021K0ZF, 021K0ZW, 021K4Z8, 021K4Z9, 021K4ZC, 021K4ZF, 021K4ZW, 021L09P, 021L09Q, 021L09R, 021L0AP, 021L0AQ, 021L0AR, 021L0JP, 021L0JQ, 021L0JR, 021L0KP, 021L0KQ, 021L0KR, 021L0Z8, 021L0Z9, 021L0ZC, 021L0ZF, 021L0ZP, 021L0ZQ, 021L0ZR, 021L49P, 021L49Q, 021L49R, 021L4AP, 021L4AQ, 021L4AR, 021L4JP, 021L4JQ, 021L4JR, 021L4KP, 021L4KQ, 021L4KR, 021L4Z8, 021L4Z9, 021L4ZC, 021L4ZF, 021L4ZP, 021L4ZQ, 021L4ZR, 02700ZZ, 02703ZZ, 02704ZZ, 02710ZZ, 02713ZZ, 02714ZZ, 02720ZZ, 02723ZZ, 02724ZZ, 02730ZZ, 02733ZZ, 02734ZZ, 02C00ZZ, 02C03ZZ, 02C04ZZ, 02C10ZZ, 02C13ZZ, 02C14ZZ, 02C20ZZ, 02C23ZZ, 02C24ZZ, 02C30ZZ, 02C33ZZ, 02C34ZZ, 3E07017, 3E070PZ, 3E07317, 3E073PZ; NHI procedure code: 68023B, 68024B, 68025B, 68053B, 68054B, 68055B, 3076B, 33077B, 33078B, N26002, N26003
Cardiomyopathy	ICD-9-CM code: 425; ICD-10-CM code: I42, I43
Venous thromboembolism	ICD-9-CM code: 415.1, 451-453; ICD-10-CM code: I80-I82
Pulmonary disease	
Asthma	ICD-9-CM code: 493; ICD-10-CM code: J45
COPD	ICD-9-CM code: 491, 492, 496; ICD-10-CM code: J41-J44
Pneumonia	ICD-9-CM code: 480-486; ICD-10-CM code: J12-18
Mental disease	
Depression	ICD-9-CM code: 296.2, 296.3, 300.4, 311; ICD-10-CM code: F32-33, F34.1
Anxiety	ICD-9-CM code: 300; ICD-10-CM code: F40, F41
Schizophrenia	ICD-9-CM code: 295; ICD-10-CM code: F20, F25
Neurologic disorders	
Dementia	ICD-9-CM code: 290, 331; ICD-10-CM code: G30, F00, F01, F03, F05.1, G31.1, G31.82, G31.9
Epilepsy	ICD-9-CM code: 345, ICD-10-CM code: G40
Bone and joint disorders	
Fracture	ICD-9-CM code: 800-829; ICD-10-CM code: S12, S22, S32, S42, S52, S62, S72, S82, S92, T02, T08, T10, T12
Osteoporosis	ICD-9-CM code: 733.0; ICD-10-CM code: M81.0
Osteoarthritis	ICD-9-CM code: 715; ICD-10-CM code: M15-M19
Anemia	ICD-9-CM code: 280-285; ICD-10-CM code: D46.1, D46.4, D50-D64
Thyroid disease	ICD-9-CM code: 240-246; ICD-10-CM code: E00-E07, E35, E89.0
Chronic liver disease	ICD-9-CM code: 571; ICD-10-CM code: K70-76
Chronic renal disease	ICD-9-CM code: 250.4, 274.1, 283.11, 403.1, 404.2, 404.3, 440.1, 442.1, 447.3, 572.3, 580-588, 642.1, 646.2; ICD-10-CM code: E10.2, E11.2, E13.2, E14.2, I12, I13, N08, N18, N19
Obesity or weight gain	ICD-9-CM code: 278, V85.3, V85.4; ICD-10-CM code: E65-68
Tobacco	ICD-9-CM code: 305.1; ICD-10-CM code: F17.2
Alcohol-related disorder	ICD-9-CM code: 291, 303, 305.0, ICD-10-CM code: F10
Hypokalemia	ICD-9-CM code: 276.8; ICD-10-CM code: E87.6
Hypoglycemia	ICD-9-CM code: 251.0, 251.1, 251.2, and 250.8 (ICD-9-CM code 250.8 accompanied by the following diagnosis codes that represent other diseases were excluded: 259.8, 272.7, 681, 682, 686.9, 707, 709.3, 730.0, 730.1, 730.2, and 731.8); ICD-10-CM code: E08.641, E08.649, E09.641, E09.649, E11.63, E11.641, E11.649, E13.63, E13.641, E13.649, E14.63, E15, E16.0, E16.1, E16.2
Autoimmune disease	ICD-9-CM code: 099.3, 135, 136.1, 255.4, 287.31, 335.2, 340, 358.0, 374.53, 379.0, 390-392, 393-398, 416, 446.0, 446.4-5, 555, 556.9, 576.1, 579.0, 695.2, 695.4, 696.0-1, 701.0; ICD-10-CM code: D086, D69.3, D69.4, E27.1-E27.6, E89.6, G12.2, G12.8, G35, G70.0, H02.73, H15.0, H15.1, I00-I02, I05-I09, I27, K50, K51.9, K83.0, K90.0, L40, L52, L90.0, L93, L94.0, L94.1, L94.3, M02.30, M30, M31.3, M31.5-M31.7, M35.2
Acquired immunodeficiency syndrome	ICD-9-CM code: 042; ICD-10-CM code: B20
Cancer	ICD-9-CM code: 140-208, 230-234; ICD-10-CM code: C00-97
**Co-medications**	**Individual drugs**
Cardiovascular medications	
Angiotensin-converting enzyme inhibitors	Benazepril, captopril, cilazapril, enalapril, fosinopril, imidapril, lisinopril, perindopril, quinapril, and ramipril
Angiotensin receptor blockers	Azilsartan, candesartan, eprosartan, irbesartan, losartan, olmesartan, telmisartan, and valsartan
α-blockers	Prazosin, and doxazosin

© 2022 Wang MT et al. *JAMA Network Open.*

β-blockers	Acebutolol, alprenolol, atenolol, betaxolol, bisoprolol, bupranolol, carvedilol, esmolol, metipranolol metoprolol, labetalol, levobunolol, nadolol, oxprenolol, pindolol, propranolol, sotalol, and timolol
Calcium channel blockers	
Dihydropyridines	Amlodipine, barnidipine, benidipine, felodipine, isradipine, lacidipine, lercanidipine, nicardipine, nifedipine, nimodipine, nisoldipine, and nitrendipine
Non-dihydropyridines	Diltiazem, and verapamil
Diuretics	
Thiazides	Amiloride, bendroflumethiazide, benzylhydrochlorothiazide, clofenamide, clopamide, cyclopenthiazide, hydrochlorothiazide, hydroflumethiazide, indapamide, metolazone, , thiabutazide, and trichlormethiazide
Loop	Bumetanide, ethacrynic acid, and furosemide,
Potassium-sparing agents	Eplerenone, potassium canrenoate, spironolactone, and triamterene
Antiplatelets	Abciximab, aspirin (≤325mg/day), cilostazol, clopidogrel, dipyridamole, epoprostenol, eptifibatide, iloprost, ticagrelor, ticlopidine, tirofiban, and treprostinil
Anticoagulants	Apixaban, dabigatran, dalteparin, drotrecogin alfa, enoxaparin, fondaparinux, heparin, nadroparine, phenindione, protein C, rivaroxaban, streptokinase, tenecteplase, tinzaparin, urokinase, and warfarin
Lipid-lowering agents	
Statins	Atorvastatin, fluvastatin, lovastatin, pitavastatin, pravastatin, rosuvastatin, and simvastatin
Others	Acipimox, bezafibrate, cholestyramine, clofibrate, colestipol, etofibrate, ezetimibe, fenofibrate, gemfibrozil, niacin, niceritrol, nicofuranose, nicomol, probucol, and simfibrate
Nitrates	Glyceryl trinitrate, isosorbide dinitrate, isosorbide 5-mononitrate, and pentaerythritol tetranitrate
Antiarrhythmic agents	Adenosine, amiodarone, disopyramide, dronedarone, flecainide, lidocaine, mexiletine, prajmaline, procainamide, propafenone and quinidine
Digoxin	Digoxin
Anti-inflammatory agents	
NSAIDs	Aceclofenac, acemetacin, alclofenac, alminoprofen, aspirin(>325mg/day), auranofin, benzydamine, celecoxib, diclofenac, etodolac, etofenamate, etoricoxib, fenbufen, fenoprofen, flufenamic acid, flurbiprofen, ibuprofen, indomethacin, ketoprofen, ketorolac lysine acetylsalicylate, meclofenamic acid, mefenamic acid, meloxicam, mepirizole, nabumetone, naproxen, nefopam, niflumic acid, nimesulide, penicillamine, phenylbutazone, piroxicam, rofecoxib, salsalate, sulindac, tenoxicam, tiaprofenic acid, tiaramide, tolfenamic acid, and tolmetin
Steroids	Betamethasone, cortisone, dexamethasone, fludrocortisones, fluocortolone, hydrocortisone, methandrostenolone, methylpredisolone, nandrolone, oxymetholone, paramethasone, , prednisolone, and triamcinolone
K^+ channel opener	Nicorandil
Inhibitors of mitochondrial permeability transition pore	Cyclosporin A, Adenosine, Alfentanil, apomorphine, buprenorphine, codeine, fentanyl, hydromorphone, meperidine, morphine, noscapine, opium, tramadol
PPIs	Esomeprazole, lansoprazole, omeprazole, and pantoprazole
Anticonvulsants	Carbamazepine, clonazepam, gabapentin, lamotrigine, levetitacetam, oxcarbazepine, phenobarbital, phenytoin, pregabalin, primidone, tiagabine, topiramate, valproic acid, vigabatrin, zonisamide
Antidepressants	Agomelatine, amitriptyline, bupropion, chlordiazepoxide, citalopram, clomipramine, dothiepin, doxepin, duloxetine, escitalopram, fluoxetine, fluvoxamine, imipramine, maprotiline, melitracen, mianserin, milnacipran, mirtazapine, moclobemide, paroxetine, sertraline, trazodone, venlafaxine, viloxazine
Antipsychotics	Amisulpride, aripiprazole, chlorpheniramine, chlorpromazine, chlorprothixene, cloapine, clopenthixol, clothiapine, clotiapine, clozapine, droperidol, flupertixol, fluphenazine, haloperidol, levomepromazine, loxapine, methotrimeprazine, moperone, olanzapine, paliperidone, penfluridol, perphenazine, pimozide, pipotiazine, prochlorperazine, quetiapine, risperidone, sulpiride, thioridazine, thiothixene, tiotixene, trifluoperazine, ziprasidone, zotepine, zuclopenthixol

Abbreviations: COPD, chronic obstructive pulmonary disease; PPIs, proton pump inhibitors; NSAIDs, nonsteroidal anti-inflammatory drugs

© 2022 Wang MT et al. *JAMA Network Open.*

eTable 2. The Mean Duration and the Reasons for Truncation During Follow-Up for Add-On Mitochondrial K_{ATP} Channel High-Affinity and Low-Affinity Sulfonylurea Groups, by Outcomes

**Outcomes**	**3-point MACE***		**Myocardial infarction**		**Stroke**		**Cardiovascular death**	
	**MitoK_{ATP} channel- high affinity sulfonylureas (n = 53,714)**	**MitoK_{ATP} channel- low affinity sulfonylureas (n = 53,714)**	**MitoK_{ATP} channel- high affinity sulfonylureas (n = 53,714)**	**MitoK_{ATP} channel- low affinity sulfonylureas (n = 53,714)**	**MitoK_{ATP} channel- high affinity sulfonylureas (n =53,714)**	**MitoK_{ATP} channel- low affinity sulfonylureas (n = 53,714)**	**MitoK_{ATP} channel- high affinity sulfonylureas (n =53,714)**	**MitoK_{ATP} channel- low affinity sulfonylureas (n = 53,714)**
**Follow-up time,** mean±SD (month)	10.2 ±16.3	14.0 ±20.5	10.2 ±16.3	14.1 ±20.5	10.2 ±16.3	14.0 ±20.5	10.2 ±16.3	14.1 ±20.5
**Follow-up time,** median (interquartile, month)	3.8 (1.7, 10.7)	5.2 (2.1, 16.3)	3.8 (1.7, 10.7)	5.2 (2.1, 16.4)	3.8 (1.7, 10.7)	5.2 (2.1, 16.3)	3.8 (1.7, 10.7)	5.2 (2.1, 16.4)
**Censorship, %**								
Discontinuation	54.1	59.0	54.3	59.2	54.2	59.0	54.3	59.2
Switching	29.7	24.7	29.9	25.0	29.8	24.8	30.0	25.1
Add-on[†]	11.3	8.4	11.4	8.5	11.4	8.5	11.5	8.6
Pregnancy	0.1	0.1	0.1	0.1	0.1	0.1	0.1	0.1
Disenrollment	0.4	0.6	0.4	0.7	0.4	0.7	0.4	0.6
End of study	3.6	6.3	3.7	6.3	3.7	6.3	3.7	6.3
Outcomes	0.8	0.9	0.2	0.2	0.6	0.6	0.1	0.1

© 2022 Wang MT et al. *JAMA Network Open.*

Outcomes	Arrhythmia		Heart failure		All-cause mortality		Severe hypoglycemia	
	**MitoK_{ATP} channel-high affinity sulfonylureas (n = 53,714)**	**MitoK_{ATP} channel-low affinity sulfonylureas (n = 53,714)**	**MitoK_{ATP} channel-high affinity sulfonylureas (n = 53,714)**	**MitoK_{ATP} channel-low affinity sulfonylureas (n = 53,714)**	**MitoK_{ATP} channel-high affinity sulfonylureas (n =53,714)**	**MitoK_{ATP} channel-low affinity sulfonylureas (n = 53,714)**	**MitoK_{ATP} channel-high affinity sulfonylureas (n =53,714)**	**MitoK_{ATP} channel-low affinity sulfonylureas (n = 53,714)**
**Follow-up time,** mean±SD (month)	10.2 ±16.3	14.0 ±20.5	10.2 ±16.3	14.1 ±20.5	10.2 ±16.3	14.1 ±20.5	10.1 ±16.3	14.0 ±20.5
**Follow-up time,** median (interquartile, month)	3.8 (1.7, 10.7)	5.2 (2.1, 16.4)	3.8 (1.7, 10.7)	5.2 (2.1, 16.4)	3.8 (1.7, 10.7)	5.2 (2.1, 16.4)	3.8 (1.7, 10.7)	5.2 (2.1, 16.3)
**Censorship, %**								
Discontinuation	54.3	59.2	54.3	59.2	54.3	59.2	53.9	59.0
Switching	29.9	25.0	30.0	25.1	30.0	25.1	29.8	25.0
Add-on[†]	11.5	8.6	11.5	8.6	11.5	8.6	11.3	8.4
Pregnancy	0.1	0.1	0.1	0.1	0.1	0.1	0.1	0.1
Disenrollment	0.4	0.7	0.4	0.7	0.2	0.4	0.4	0.7
End of study	3.7	6.3	3.7	6.3	3.7	6.3	3.7	6.3
Outcomes	0.2	0.2	0.1	0.1	0.3	0.3	0.9	0.6

Abbreviations: Mito, mitochondrial; MACE, major adverse cardiovascular events; SD, standard deviation.

[*]3-point MACE includes myocardial infarction, ischemic stroke, and cardiovascular death.

[†]Add-on represents the addition of other antidiabetic agents.

© 2022 Wang MT et al. *JAMA Network Open.*

eTable 3. Number Needed to Harm for Add-On Mitochondrial K_{ATP} Channel High-Affinity and Low-Affinity Sulfonylureas In Patients Continuously Receiving Metformin Monotherapy

	MitoK_{ATP} channel-high affinity sulfonylureas (n=53,714)	MitoK_{ATP} channel-low affinity sulfonylureas (n=53,714)	
	Incident rate / 100 person-year (95% CI)	Incident rate / 100 person-year (95% CI)	NNH[a]
3-point MACE	0.99 (0.90-1.09)	0.78 (0.72-0.86)	476
Myocardial infarction	0.26 (0.22-0.31)	0.19 (0.16-0.23)	1429
Ischemic stroke	0.66 (0.59-0.74)	0.53 (0.48-0.59)	NA
Cardiovascular death	0.08 (0.06-0.12)	0.06 (0.05-0.09)	NA
Arrhythmia	0.18 (0.15-0.22)	0.14 (0.12-0.18)	NA
Heart failure	0.13 (0.10-0.17)	0.09 (0.07-0.12)	NA
All-cause mortality	0.38 (0.32-0.44)	0.28 (0.24-0.33)	1000
Severe hypoglycemia	0.90 (0.83-0.98)	0.61 (0.54-0.69)	345

Abbreviations: Mito, mitochondrial; NNH, number needed to harm; MACE, major adverse cardiovascular events; CI, confidence interval

[a]NNH were calculated by using the following formula: absolute risk reduction (ARR) = (the event rate of mitoK_{ATP} channel-high affinity sulfonylureas) – (the event rate of mitoK_{ATP} channel-low affinity sulfonylureas); NNH = 1 / ARR

© 2022 Wang MT et al. *JAMA Network Open.*

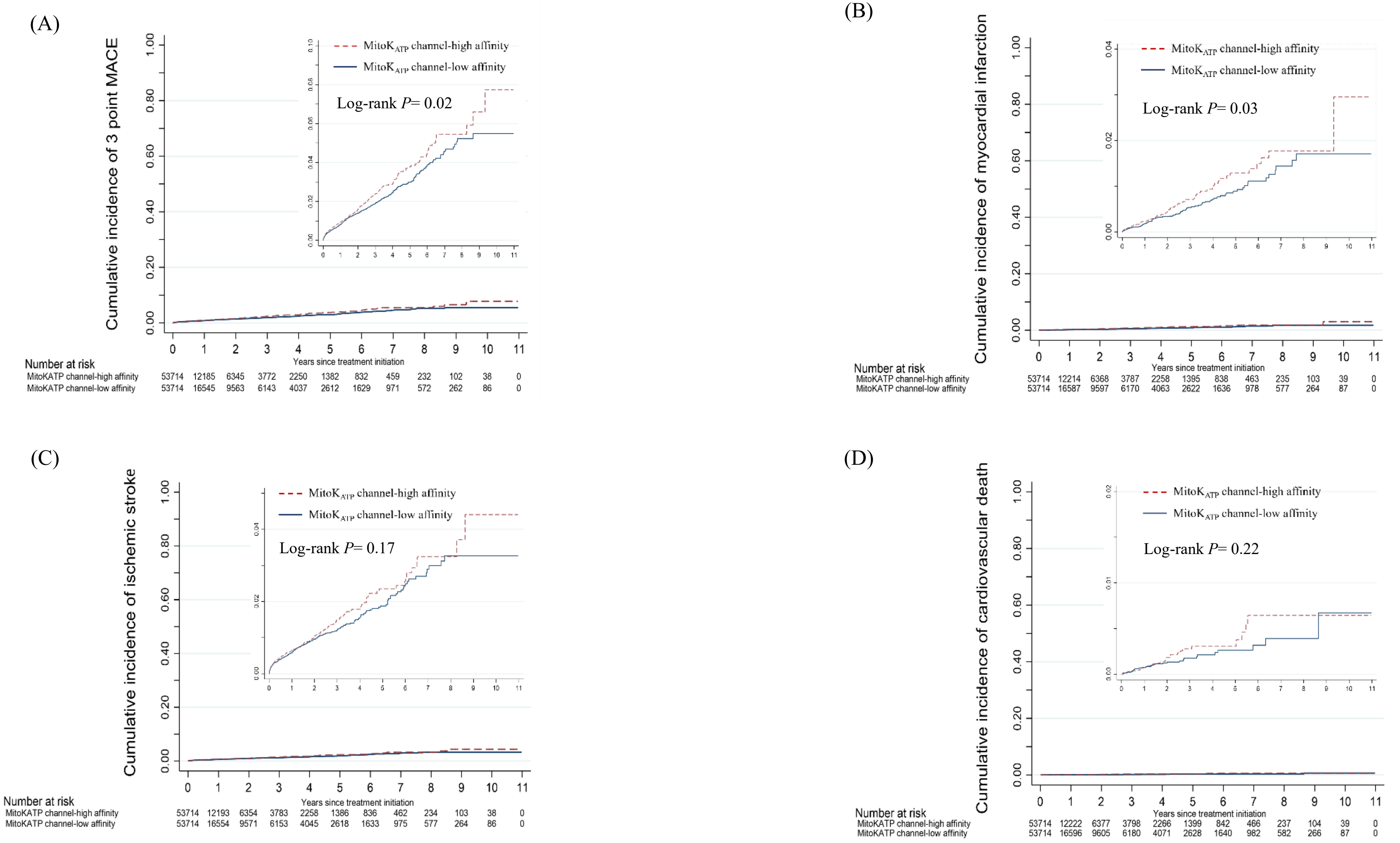

eFigure 1. Kaplan-Meier Survival Curves of 3-Point Major Adverse Cardiovascular Events (A), Myocardial Infarction (B), Ischemic Stroke (C), and Cardiovascular Death (D) Between Metformin Users With Add-On Mitochondrial K_{ATP} Channel High-Affinity Sulfonylurea and Mitochondrial K_{ATP} Channel Low-Affinity Sulfonylurea

Abbreviations: MACE, major adverse cardiovascular events; Mito, mitochondrial.

© 2022 Wang MT et al. *JAMA Network Open.*

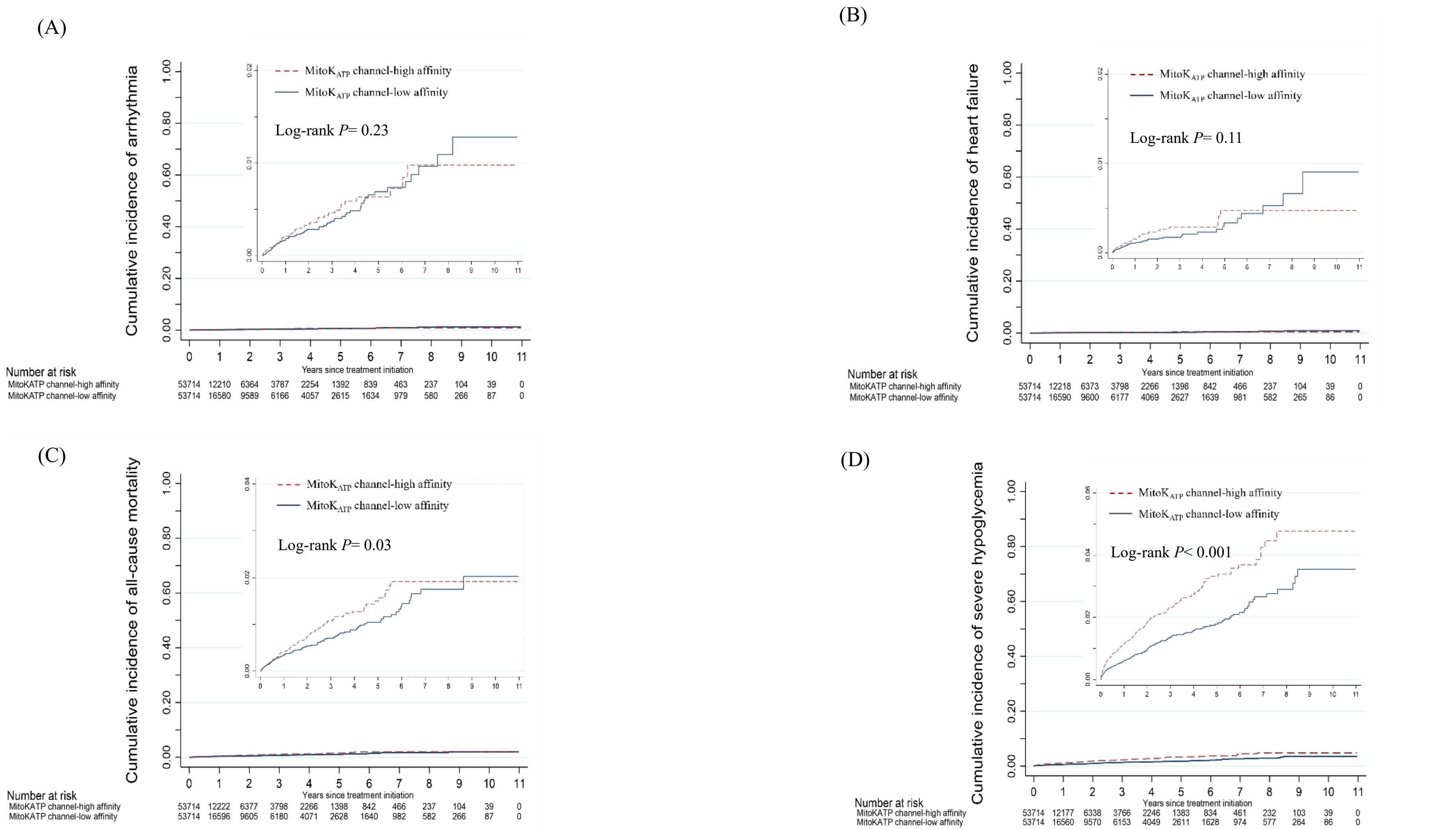

eFigure 2. Kaplan-Meier Survival Curves of Arrhythmia (A), Heart Failure (B), All-Cause Mortality (C), and Severe Hypoglycemia (D) Between Metformin Users With Add-On Mitochondrial K_{ATP} Channel High-Affinity Sulfonylurea and Mitochondrial K_{ATP} Channel Low-Affinity Sulfonylurea

Abbreviations: Mito, mitochondrial.

© 2022 Wang MT et al. *JAMA Network Open.*

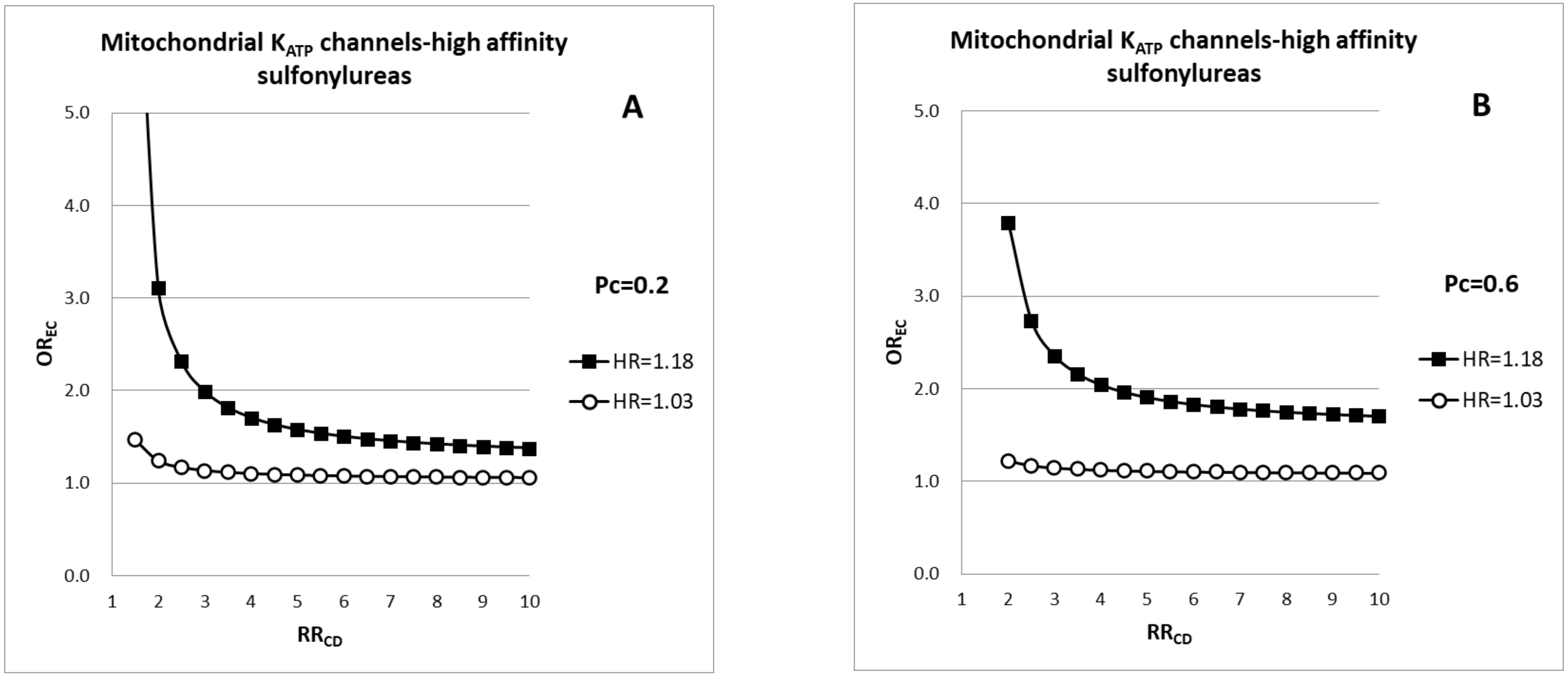

eFigure 3. Employment of the Rule-Out Approach to Assess the Impact of Unmeasured Confounding on the Main Findings

For the estimations, the prevalence of mitochondrial K_{ATP} channel-high affinity sulfonylureas was estimated to be 22%, and we carried out the analyses assuming that the prevalence of an unmeasured confounder was 20% and 60% in panels A and B, respectively. In all panels, the combinations of RR_{CD} and OR_{EC} either lie in the upper right zone of each line or on the line, indicating confounding effect could contribute to the observed increased rate of cardiovascular adverse events associated with mitochondrial K_{ATP} channel-high affinity sulfonylureas versus mitochondrial K_{ATP} channel-low affinity sulfonylureas among diabetic patients using metformin monotherapy. HR = hazard ratio; RR_{CD} = relative risk of cardiovascular adverse events with an unmeasured confounder; OR_{EC} = odds ratio representing the association between use of mitochondrial K_{ATP} channel-high affinity sulfonylureas (versus use of mitochondrial K_{ATP} channel-low affinity sulfonylureas) and an unmeasured confounder; P_c = the prevalence of an unmeasured confounder.

© 2022 Wang MT et al. *JAMA Network Open.*
