# Supplementary material for: Comparison of Mitochondrial Adenosine Triphosphate–Sensitive Potassium Channel High- vs Low-Affinity Sulfonylureas and Cardiovascular Outcomes in Patients With Type 2 Diabetes Treated With Metformin
Source: JAMA Netw Open. 2022 Dec 9;5(12):e2245854. doi: 10.1001/jamanetworkopen.2022.45854 (PMC9856426; doi:10.1001/jamanetworkopen.2022.45854)
Supplement: Supplement 2. — Data Sharing Statement [file jamanetwopen-e2245854-s002.pdf]

Data Sharing Statement

Wang. Comparison of Mitochondrial Adenosine Triphosphate-Sensitive Potassium Channel High- vs Low-Affinity Sulfonylureas and Cardiovascular Outcomes in Patients With Type 2 Diabetes Treated With Metformin. *JAMA Netw Open*. Published December 09, 2022. doi:10.1001/jamanetworkopen.2022.45854

Data
Data available: No

Additional Information
Explanation for why data not available: This study primarily utilized claims data from the Health and Welfare Data Source Center, Ministry of Health and Welfare (HWDC, MOHW), Taiwan. The access to the analyzed database needs the approval from the HWDC, MOHW. Additionally, all analyses were performed at the HWDC on site, and any individual-level data were not allowed to be taken out.
